# Shift a laser beam back and forth to exchange heat and work in thermodynamics

**DOI:** 10.1038/s41598-021-83824-7

**Published:** 2021-02-23

**Authors:** John A. C. Albay, Zhi-Yi Zhou, Cheng-Hung Chang, Yonggun Jun

**Affiliations:** 1 Department of Physics, National Central University, Taoyuan, 320 Taiwan; 2 Institute of Physics, National Chiao Tung University, Hsinchu, 300 Taiwan

**Keywords:** Statistical physics, thermodynamics and nonlinear dynamics, Techniques and instrumentation

## Abstract

Although the equivalence of heat and work has been unveiled since Joule’s ingenious experiment in 1845, they rarely originate from the same source in experiments. In this study, we theoretically and experimentally demonstrated how to use a high-precision optical feedback trap to combine the generation of virtual temperature and potential to simultaneously manipulate the heat and work of a small system. This idea was applied to a microscopic Stirling engine consisting of a Brownian particle under a time-varying confining potential and temperature. The experimental results justified the position and the velocity equipartition theorem, confirmed several theoretically predicted energetics, and revealed the engine efficiency as well as its trade-off relation with the output power. The small theory–experiment discrepancy and high flexibility of the swift change of the particle condition highlight the advantage of this optical technique and prove it to be an efficient way for exploring heat and work-related issues in the modern thermodynamics for small systems.

## Introduction

Heat and work are two core quantities in thermodynamics, a good control of which is decisive for how broadly we can explore this field. The most typical system related to the exchange of heat and work might be the heat engine. This old issue has been a relatively complete chapter in the traditional thermodynamics of the macroscopic world^1^ and gained a wide application in industry^2^. However, due to the recent advance in the theory of small systems^3–6^ and the technology for manipulating microscopic objects^7–9^, there has been a renewed interest in that old issue, but this time with a focus shifted to the microscopic scale. Nowadays, examples for microscopic heat engines have covered a wide spectrum, ranging from those as classical as the microscopic Stirling heat engine^10,11^ and the Brownian Carnot heat engine^12^ to those as versatile as the microscopic steam engine^13^, the Brownian gyrator^14,15^, the microscopic rotary engine^16^, and the single atom engine^17^.

Among the existing cyclic microscopic heat engines, a paradigm model is a Brownian particle under a cyclic variation of confining potential and temperature. This miniature system plays the same role as its macroscopic counterpart of the piston-cylinder model for understanding heat engines and has been experimentally implemented in different ways. For instance, Blickle *et al.* have optically trapped the particle and heated up the surrounding medium to vary the real temperature^10^. Martinez *et al.* have applied a noisy electrostatic force to generate an artificial temperature to replace the above real temperature^12^. In all those experiments, a common feature is that the temperature and the confining potential are separately prepared. It reflects the fundamental difference between heat and work, distinguished by whether the transferred energy is ordered or disordered. In contrast to these conventional operations, this work combines the generation of temperature and confining potential by using a single laser beam of constant intensity in the technique of optical feedback trap (OFT) and applies it to realize a microscopic heat engine.

The key idea behind this technique is that to let a Brownian particle perceive a potential, *U*(*x*), it is sufficient to generate a force *f*(*x*, *t*) on that particle which satisfies $f(x(t),t)=[-dU(x')/dx']_{x'=x(t)}$ only at the instant location, *x*(*t*), of the particle^18,19^. Since the particle is moving, *f*(*x*, *t*) needs to change swiftly to match the above equality at any time *t*. A simple choice for such a feedback force is the force of an optical tweezers of constant intensity, whose magnitude on the particle is tuned by relocating the center of the tweezers. The effective potential $U_{\mathrm{v}}(x)$ generated by this feedback force to approach the real potential *U*(*x*) is termed a virtual potential (VP). $U_{\mathrm{v}}(x)$ will be close to *U*(*x*) if one can precisely detect the position of the Brownian particle and has an ultrafast feedback system to tune the force, *f*(*x*, *t*), which has been shown feasible^20,21^. If the time-independent *U*(*x*) is replaced by a time-dependent potential *U*(*x*, *t*) of an arbitrary shape controlled by some protocol, the strategy to determine its *f*(*x*, *t*) is the same. If an extra fluctuation is added to *f*(*x*, *t*), this force will additionally give a virtual temperature (VT) to the particle. Owing to this relation, one should be able to simultaneously control the VP and VT of a stochastic thermodynamic process merely by properly shifting the center of a laser beam.

To examine its applicability, we first used the feedback force to create a virtual system (VP and VT) to mimic the environment of a Brownian particle confined in a real harmonic potential at a real temperature. The stiffnesses of that potential and the temperatures inferred from the position (acquisition-rate-dependent velocity) of that particle via the position (modified velocity) equipartition theorem were shown to highly coincide with those assigned to the experiment. When the stiffness and temperature were varied, the cumulative sums of heat, work, potential energy change, and kinetic energy change agreed well with those given by the analytical formulas. Based on this consistency, we built up a microscopic Stirling engine and determined the trade-off relation between its efficiency and output power, as well as the efficiency at maximum power $\eta ^*\approx 0.24$. The outcomes confirmed several theoretical predictions^22,23^ and experimental trends observed in other small systems^10,12,20,24^ (see Figs. 2, 3, 4, 5, 6). In comparison with previous experiments, the instant particle response and temperature change by using the OFT reduces the theory–experiment discrepancy in the calculation of kinetic energy and extends the observation capability far beyond the quasi-static regime. It indicates that steering a laser beam to realize a microscopic engine is not only possible but even more accurate. This suggests the OFT to be a promising technique for studying general heat-work exchange problems in stochastic thermodynamics.

## Methods

### The theory and experimental setup for the optical feedback trap

**Figure 1:**
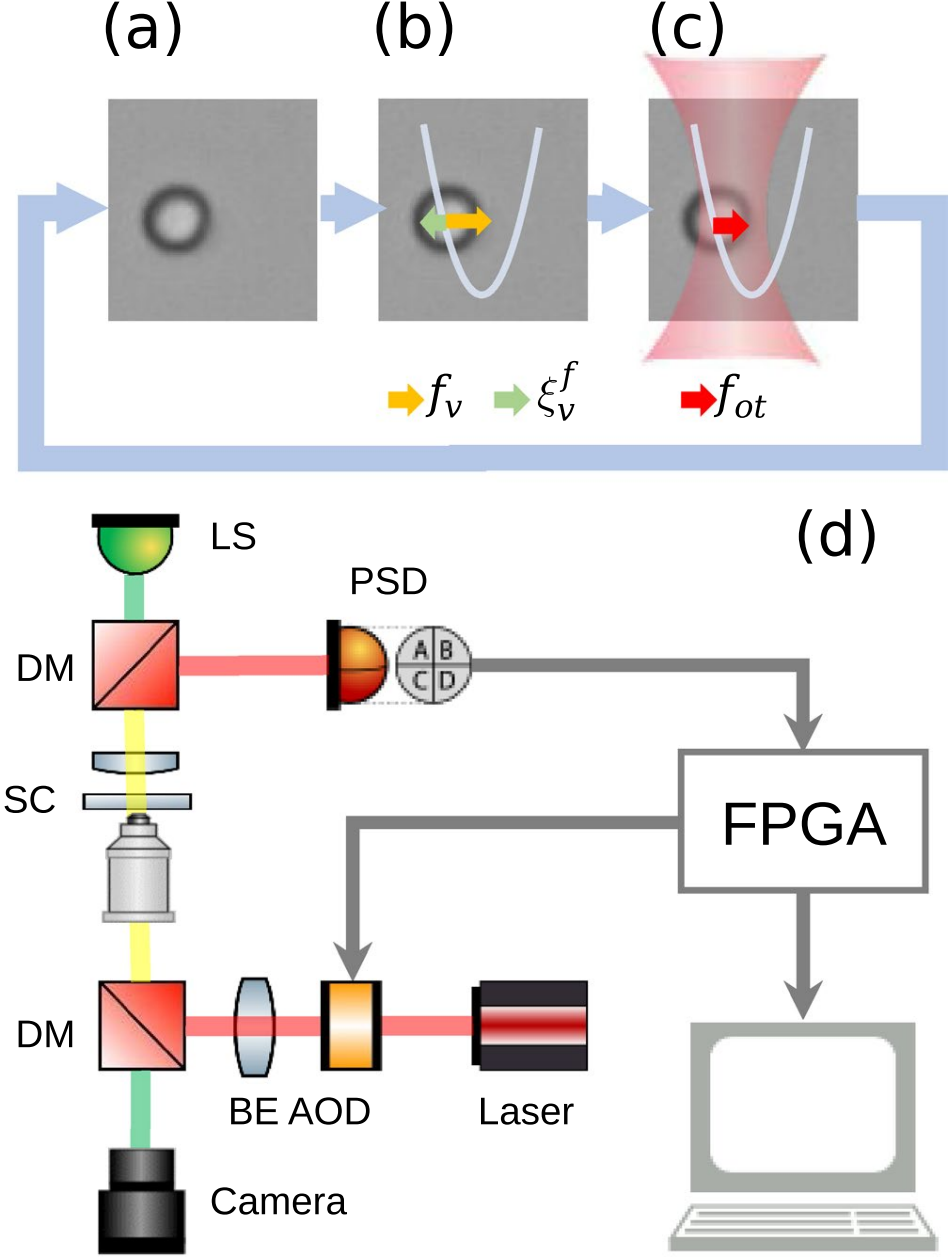
(**a**)–(**c**) The feedback protocol for generating the VP and VT. (**a**) Identify the particle position using the PSD. (**b**) Compute the force, $f_{\mathrm{v}}+\xi_{\mathrm{v}}^{\mathrm{f}}$, required to generate the effects of the assigned VP and VT. (**c**) Deflect the laser center by the AOD to apply the same amount of force, $f_{\mathrm{ot}}=f_{\mathrm{v}}+\xi_{\mathrm{v}}^{\mathrm{f}}$, on the particle. (**d**) A schematic drawing of the OFT. DM: dichroic mirror, PSD: position sensitive device, AOD: acousto-optic deflector, FPGA: Field programmable gate array, LS: light source, BE: beam expander, and SC: sample cell.

Let *x*(*t*) be the position of a freely moving one-dimensional Brownian particle in a surrounding medium at time *t* (Fig. 1a). The particle is subject to the thermal noise $\xi ^{\mathrm{f}}$ and an external noise $\xi_{\mathrm{v}}^{\mathrm{f}}$ (green arrow in Fig. 1b), which are Gaussian and white, with $\langle \xi ^{\mathrm{f}}(t)\rangle =\langle \xi_{\mathrm{v}}^{\mathrm{f}}(t)\rangle =\langle \xi ^{\mathrm{f}}(t)\xi_{\mathrm{v}}^{\mathrm{f}}(t')\rangle =0$, $\langle \xi ^{\mathrm{f}}(t)\xi ^{\mathrm{f}}(t')\rangle =2\gamma k_{\mathrm{B}} T_{\mathrm{R}}\delta (t-t')$, and $\langle \xi_{\mathrm{v}}^{\mathrm{f}}(t)\xi_{\mathrm{v}}^{\mathrm{f}}(t')\rangle =2\gamma k_{\mathrm{B}} T_{\mathrm{v}}\delta (t-t')$. Here $k_{\mathrm{B}}$ denotes the Boltzmann constant, $\delta (t-t')$ represents the Dirac delta function, and $T_{\mathrm{R}}$ and $T_{\mathrm{v}}$ stand for the room temperature and a virtual temperature, respectively. Thus, these stochastic forces will be given by $\xi ^{\mathrm{f}}=\sqrt{2\gamma k_{\mathrm{B}}T_{\mathrm{R}}} g(1)$ and $\xi_{\mathrm{v}}^{\mathrm{f}}=\sqrt{2\gamma k_{\mathrm{B}}T_{\mathrm{v}}} g(1)$, respectively, with *g*(1) a Gaussian random noise of zero mean and unit variance. We address that the friction coefficient $\gamma$ in $\xi_{\mathrm{v}}^{\mathrm{f}}$ is chosen to be the same as that in $\xi ^{\mathrm{f}}$ as usual, to keep the artificial temperature $T_{\mathrm{v}}$ (an energy scale) given by $\xi_{\mathrm{v}}^{\mathrm{f}}$ simple. If we impose a harmonic VP, $U_{\mathrm{v}}(x)=(1/2)k_{\mathrm{v}}x^{2}$ to confine the particle, the latter will experience a restoring force $f_{\mathrm{v}}=-k_{\mathrm{v}}x$ (yellow arrow in Fig. 1b), with $k_{\mathrm{v}}$ the stiffness of the VP. We stress that $\xi_{\mathrm{v}}^{\mathrm{f}}$ and $f_{\mathrm{v}}$ are not the real forces given by the genuine quantities of temperature and potential, but the artificial ones generated by the OFT.

In this experiment, the motion of the Brownian particle is in the overdamped regime, which follows the Langevin equation $\gamma {\dot{x}}-f_{\mathrm{v}}-\xi_{\mathrm{v}}^{\mathrm{f}}=\xi ^{\mathrm{f}}$. If the data acquisition frequency to trace the particle is $f_{\mathrm{u}}<\infty$, only discrete particle positions $...,\,x_t,\,x_{t+t_{\mathrm{u}}},\,x_{t+2t_{\mathrm{u}}},\,x_{t+3t_{\mathrm{u}}},...$ will be recorded, where $t_{\mathrm{u}}=1/f_{\mathrm{u}}$ is the data acquisition time. This discrete dynamics will satisfy a difference equation $\gamma (x_{t+t_{\mathrm{u}}}-x_t)/t_{\mathrm{u}}-{\tilde{f}}_{\mathrm{v}}-{\tilde{\xi }}_{\mathrm{v}}^{\mathrm{f}}={\tilde{\xi }}^{\mathrm{f}}$, with ${\tilde{f}}_{\mathrm{v}}=-k_{\mathrm{v}}x_t$, ${\tilde{\xi }}_{\mathrm{v}}^{\mathrm{f}}\equiv (\gamma /t_{\mathrm{u}})\sqrt{k_{\mathrm{B}}T_{\mathrm{v}}(1-\Omega ^2)/k_{\mathrm{ot}}} g(1)$, and ${\tilde{\xi }}^{\mathrm{f}}\equiv (\gamma /t_{\mathrm{u}})\sqrt{k_{\mathrm{B}}T_{\mathrm{R}}(1-\Omega ^2)/k_{\mathrm{ot}}} g(1)$, where $\Omega \equiv 1-t_{\mathrm{u}}/\tau _R$ and $\tau _R\equiv \gamma /k_{\mathrm{v}}$ is the relaxation time of the particle in the VP (see Supplemental Material). This form of ${\tilde{\xi }}_{\mathrm{v}}^{\mathrm{f}}$ and ${\tilde{\xi }}^{\mathrm{f}}$ will consistently yield an identical mean squared displacement of the particle dynamics for different small $t_{\mathrm{u}}$, irrespective of whether the particle is in a free space ($k_{\mathrm{v}}=0$) or a confined space ($k_{\mathrm{v}}>0$). For $t_{\mathrm{u}}\ll \tau _{\mathrm{R}}$, ${\tilde{\xi }}_{\mathrm{v}}^{\mathrm{f}}$ and ${\tilde{\xi }}^{\mathrm{f}}$ will reduce to the simple form $\sqrt{2\gamma k_{\mathrm{B}}T_{\mathrm{v}}/t_{\mathrm{u}}}g(1)$ and $\sqrt{2\gamma k_{\mathrm{B}}T_{\mathrm{R}}/t_{\mathrm{u}}}g(1)$, respectively, as in the current experiment, where $t_{\mathrm{u}}$ is 100 times smaller than $\tau _{\mathrm{R}}$.

In the above difference equation, the forces ${\tilde{f}}_{\mathrm{v}}$ and ${\tilde{\xi }}_{\mathrm{v}}^{\mathrm{f}}$ account for the effects of $U_{\mathrm{v}}$ and $T_{\mathrm{v}}$ to be experienced by the particle. Their amounts can be summed up as a net value and provided by the force of an optical tweezers, $f_{\mathrm{ot}}=-k_{\mathrm{ot}}(x_t-x_{\mathrm{L},t})={\tilde{f}}_{\mathrm{v}}+{\tilde{\xi }}_{\mathrm{v}}^{\mathrm{f}}$ (red arrow in Fig. 1c), with $x_{\mathrm{L},t}$ the center position and $k_{\mathrm{ot}}$ the stiffness of the tweezers. Therefore, ideally a requested force amount, $f_{\mathrm{ot}}$, could be generated if we were able to shift the laser center to the position $x_{\mathrm{L},t}=-\alpha x_t+{\tilde{\xi }}_{\mathrm{v}}^{\mathrm{f}}/k_{\mathrm{ot}}$ instantaneously after the particle location $x_t$ is detected, where $\alpha \equiv -(1-k_{\mathrm{v}}/k_{\mathrm{ot}})$ is the feedback gain. However, in practice one can only achieve

1 $$\begin{aligned} x_{\mathrm{L},t}=-\alpha x_{t-t_{\mathrm{d}}}+{\tilde{\xi }}_{\mathrm{v}}^{\mathrm{f}}/k_{\mathrm{ot}}, \end{aligned}$$

with an inevitable small delay time $t_{\mathrm{d}}$ required for the calculation of the feedback force. Clearly, the dynamics of $x_t$ and $x_{\mathrm{L},t}$ are coupled to each other. Nevertheless, inserting the $x_{\mathrm{L},t}$ in Eq. (1) into the above $f_{\mathrm{ot}}$ equation and the latter into the above difference equation, one can decouple these two dynamics and obtain a difference equation purely for the evolution of the Brownian particle in the OFT (see Supplemental Material)

2 $$\begin{aligned} \gamma \frac{x_{t+t_{\mathrm{u}}}-x_t}{t_{\mathrm{u}}}+k_{\mathrm{ot}}\left( x_t+\alpha x_{t-t_{d}}\right) ={\tilde{\xi }}^{f}+{\tilde{\xi }}_{\mathrm{v}}^{\mathrm{f}}. \end{aligned}$$

Following Eq. (1), the particle would behave as if it were in a real harmonic potential of stiffness $k_{\mathrm{v}}$ at a real temperature of value $T_{\mathrm{v}}$. When the thermal noise ${\tilde{\xi }}^{f}$ is also considered, as in Eq. (2), the particle will feel itself like at the kinetic temperature $T_{\mathrm{kin}}=T_{\mathrm{R}}+T_{\mathrm{v}}$. Notably, when $T_{\mathrm{v}}$ is varied in the experiment, $\gamma$ is a constant because we do not change $T_{\mathrm{R}}$.

The experimental setup to realize the VP has been introduced in Ref.^20^, which can be generalized to include the VT effect as described above. Here we briefly sketch the setup and refer its details to Ref.^20^. The feedback control loop of the entire trapping system is depicted in Fig. 1d. The Brownian particle in the experiment is a polystyrene particle of 1 $\mu$m-diameter, which has a mass of $m=5.6\times 10^{-16}$ kg and is immersed in water. Its location, $x_t$, is detected by a position sensitive device (PSD) with a resolution of about 1 nm. This data is sent to the homemade LabVIEW program on a field-programmable gate array (FPGA) to compute the $f_{\mathrm{ot}}$ of the desired VP and VT. Once computed, that amount of force is applied to the particle using the acousto-optic deflector (AOD), which deflects the center of a laser beam of constant intensity to the position $x_{\mathrm{L},t}$ with a delay time $t_{\mathrm{d}}$ after the particle position is detected. The particle position, $x_t$, is acquired at every 10 $\mu$s, denoted by $t_{\mathrm{u}}$, and $10^5$ position data are stored every second in the FPGA before sent to the computer for further analysis. The parameter values used in the experiment are $t_{\mathrm{d}}=t_{\mathrm{u}}=10$ $\mu$s, $k_{\mathrm{ot}}=30$ pN/$\mu$m, $\gamma =1\times 10^{-8}$ kg/s, and $T_{\mathrm{R}}=300$ K. The stiffness of the laser trap is calibrated by two methods: the equipartition theorems and the power spectrum analysis^25^, as shown below.

## Results

### The position distribution and power spectrum density

**Figure 2:**
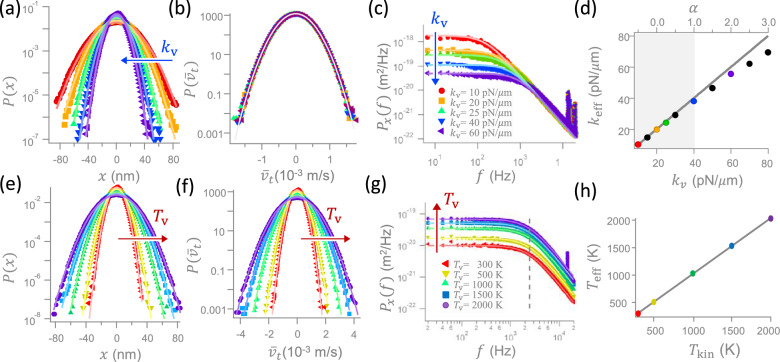
The PDFs of the position fluctuations (**a**) and of the velocity fluctuations (**b**), as well as the corresponding power spectrum densities (**c**) for an increasing virtual stiffness, $k_{\mathrm{v}}$, (blue arrow) at a fixed virtual temperature, $T_{\mathrm{v}}=0$, (or $T_{\mathrm{kin}}=T_{\mathrm{R}}$). (**d**) A comparison between the assigned stiffness, $k_{\mathrm{v}}$, and the measured stiffness, $k_{\mathrm{eff}}$. The symbols in (**a**), (**b**), and (**c**) have the same $k_{\mathrm{v}}$ values as the symbols of the same colors in (**d**). (**e**), (**f**), and (**g**) are similar to (**a**), (**b**), and (**c**), but for an increasing $T_{\mathrm{v}}$ (red arrows) at a fixed stiffness, $k_{\mathrm{v}}=k_{\mathrm{ot}}$, which corresponds to $\alpha =0$. (**h**) A comparison between the assigned temperature, $T_{\mathrm{kin}}$, and the measured temperature, $T_{\mathrm{eff}}$. The symbols in (**e**), (**f**), and (**g**) have the same $T_{\mathrm{kin}}=T_{\mathrm{v}}+T_{\mathrm{R}}$ values as the symbols of the same colors in (**h**). While the symbols in (**a**), (**b**), (**c**), (**e**), (**f**), and (**g**) are experimental values, the lines in (**a**), (**b**), (**e**) and (**f**) are fitting functions and those in (**c**) and (**g**) are theoretical functions given by Eq. (3). The slopes of the solid lines in (**d**) and (**h**) are 1.

As the first step, we investigate the probability distribution functions (PDF) of the position and the velocity of the particle. Here, the velocity along a trajectory is calculated by ${\bar{v}}_t=(x_t-x_{t-t_{\mathrm{u}}})/t_{\mathrm{u}}$, with a small $t_{\mathrm{u}}$. The analysis is carried out in two scenarios: (i) varying $k_{\mathrm{v}}$ under a fixed $T_{\mathrm{v}}$ and (ii) varying $T_{\mathrm{v}}$ under a fixed $k_{\mathrm{v}}$. For case (i), the variances of the position PDF decrease with the increasing stiffness $k_{\mathrm{v}}$ (Fig. 2a), while those of the velocity PDF remain constant (Fig. 2b). For case (ii), the variances of both position and velocity PDFs increase with $T_{\mathrm{v}}=T_{\mathrm{kin}}-T_{\mathrm{R}}$ (Fig. 2e,f, respectively). The dependence of the PDFs on $k_{\mathrm{v}}$ and $T_{\mathrm{v}}$ in these four plots shares the same feature as the PDFs under the variation of a real potential and temperature.

To inspect the fluctuations of the particle, let us further consider the power spectrum density function of the particle position under a feedback control of delay time $t_{\mathrm{d}}$^20^,

3 $$\begin{aligned} P_x(f)=\frac{k_{\mathrm{B}} T_{\mathrm{kin}}}{\pi ^2 \gamma \left| jf+f_{\mathrm{c}}+f_{\mathrm{c}} \alpha \exp {(-j2\pi ft_{\mathrm{d}} )}\right| ^2}, \end{aligned}$$

with *j* the imaginary unit. Here, $f_{\mathrm{c}}\equiv k_{\mathrm{ot}}/(2\pi \gamma )$ is the corner frequency of $P_x(f)$ at $\alpha =0$, which denotes the onset of the decline of $P_x(f)$ at that $\alpha$. In Fig. 2c,g, the experimentally measured $P_x(f)$ (symbols) for various $k_{\mathrm{v}}$ and $T_{\mathrm{v}}$, respectively, agree very well with the theoretically predicted $P_x(f)$ given by Eq. (3) (solid lines), where $T_{\mathrm{kin}}$ in that equation is related to the $T_{\mathrm{v}}$ of the symbols by $T_{\mathrm{kin}}=T_{\mathrm{R}}+T_{\mathrm{v}}$.

In Fig. 2c, all $P_x(f)$ behave like Lorentzian. However, they will gradually deform to a non-Lorentzian shape with an emerging resonance peak if $k_{\mathrm{v}}$ is further increased, in consistent with what reported before^20^. To use a VP to catch the correct effect of a sharp potential, we need to reduce $t_{\mathrm{u}}$ to a sufficiently small value to avoid this unphysical non-Lorentzian feature. By contrast, this artifact is insignificant in Fig. 2g, where all $P_x(f)$ are Lorentzian at least up to $T_{\mathrm{v}}\le 2000$ K. Since all the measured $P_x(f)$ in this plot have the same $f_{\mathrm{c}}$ (gray dashed line) and $k_{\mathrm{ot}}=k_{\mathrm{v}}$, the definition $f_{\mathrm{c}}=k_{\mathrm{ot}}/(2\pi \gamma )$ implies that an increasing noise intensity does not change the stiffness $k_{\mathrm{v}}$ of the VP, but only the VT. Notably, the tiny peaks in the high frequency regime in Fig. 2c,g are experimental noises caused by the electric background or the laser intensity fluctuations, irrelevant to the above-mentioned prominent resonance peak.

Next, we examine whether the position equipartition theorem holds in the above virtual systems. To this end, let us compare the effective stiffness $k_{\mathrm{eff}}\equiv k_{\mathrm{B}} T_{\mathrm{kin}}/\langle x^2\rangle$ (effective temperature $T_{\mathrm{eff}}\equiv k_{\mathrm{v}}\langle x^2\rangle /k_{\mathrm{B}}$) inferred from the variances of the PDFs in Fig. 2a (Fig. 2e) with the $k_{\mathrm{v}}$ ($T_{\mathrm{kin}}$) of the virtual system assigned to the experiment. In Fig. 2a,e, we have set $T_{\mathrm{kin}}=T_{\mathrm{R}}$ and $k_{\mathrm{v}}=k_{\mathrm{ot}}$, respectively. In the comparison of stiffnesses, Fig. 2d shows a coincidence between the values of $k_{\mathrm{eff}}$ and $k_{\mathrm{v}}$ up to $k_{\mathrm{v}}\approx 40$ pN/$\mu$m (gray shadowed regime), indicating that the position equipartition relation, $k_{\mathrm{v}} \langle x^2\rangle =k_{\mathrm{B}} T_{\mathrm{kin}}$, holds for the VP system at least in this stiffness regime. For $k_{\mathrm{v}}>$ ${40}{pN/\upmu \hbox {m}}$, $P_x(f)$ starts to deviate from the above-mentioned Lorentzian due to the particle overshoot^20^. As a result, the $k_{\mathrm{eff}}\equiv k_{\mathrm{B}}T_{\mathrm{kin}}/\langle x^2\rangle$ calculated from a deformed $\langle x^2\rangle$ will deviate from the assigned $k_{\mathrm{v}}$. This discrepancy can be suppressed by reducing $t_{\mathrm{d}}$. In the comparison of temperatures, Fig. 2h reveals an agreement between the values of $T_{\mathrm{eff}}$ and $T_{\mathrm{kin}}=T_{\mathrm{R}}+T_{\mathrm{v}}$ within 2000 K, which validates the same equipartition relation as above in the VT system at least up to that temperature. Alternatively, $T_{\mathrm{eff}}$ can also be determined by the height of the plateau of $P_x(f)$ at low frequencies in Fig. 2g, which rises with $T_{\mathrm{kin}}$, as that in Fig. 2h.

**Figure 3:**
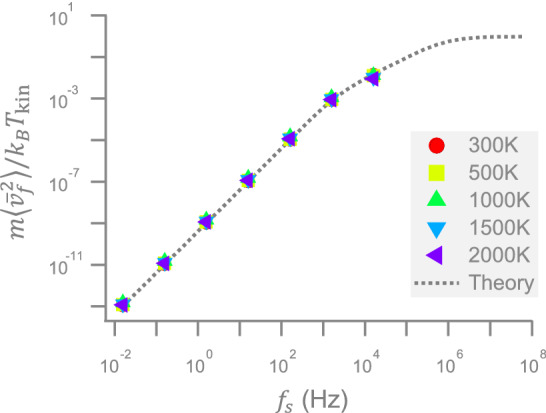
The average kinetic energy change inferred from the experimentally extracted time-averaged velocity in units of the thermal energy, $k_{\mathrm{B}}T_{\mathrm{kin}}$. The five types of symbol here have the same temperatures as those in Fig. 2e. The dashed curve is the theoretically derived kinetic energy change in Eq. (4).

The confirmed position equipartition relations in Fig. 2d,h for the position PDFs in Fig. 2a,e raise a more challenging question about whether the velocity PDFs in Fig. 2b,f also comply with the velocity equipartition relation $m\langle {\bar{v}}^2\rangle =k_{\mathrm{B}}T_{\mathrm{kin}}$, with the mean velocity ${\bar{v}}$. Here we extend the question and test $m\langle {\bar{v}}_{f_{\mathrm{s}}}^2\rangle =k_{\mathrm{B}}T_{\mathrm{kin}}$ for different mean velocities, ${\bar{v}}_{f_{\mathrm{s}}}$, averaged over a short time span $t_{\mathrm{s}}= 1/f_{\mathrm{s}}$, where the sampling frequency $f_{\mathrm{s}}$ is smaller than the above-mentioned acquisition frequency $f_{\mathrm{u}}=1/t_{\mathrm{u}}$. Notice that although *m* is absent in the overdamped formalism, we cannot neglect it in the discussion of the velocity equipartition relation, no matter how small the value of *m* is. One should not expect too much on that relation, because ${\bar{v}}_{f_{\mathrm{s}}}$ is not the instantaneous velocity. This relation is especially skeptical for a large $t_{\mathrm{s}}$, as in the current experiment with $t_{\mathrm{s}}\ge t_{\mathrm{u}}>t_{\mathrm{m}}$, where the acquisition time $t_{\mathrm{u}}=10$ $\mu$s is already much larger than the inertia time $t_m\equiv m/\gamma \approx 56$ ns.

To check the validity of the velocity equipartition relation under the above harsh condition of large $t_{\mathrm{s}}$, we calculate ${\bar{v}}_{f_{\mathrm{s}}}$ from the particle trajectories sampled at several distinct $f_{\mathrm{s}}$ between $1/2\pi$ Hz and $100/2\pi$ kHz and find rather diverse variances $\langle {\bar{v}}_{f_{\mathrm{s}}}^2\rangle$ at different $T_{\mathrm{kin}}$. However, when we consider the ratio $m\langle {\bar{v}}_{f_{\mathrm{s}}}^2\rangle /(k_{\mathrm{B}}T_{\mathrm{kin}})$, they collapse into a simple curve, as depicted by the symbols in Fig. 3. This ratio is bounded from above by 1, indicating that $\langle {\bar{v}}_{f_{\mathrm{s}}}^2\rangle$ underestimates the variance of the instantaneous velocity. That ratio will increase with $f_{\mathrm{s}}$ and tend to 1 at large $f_{\mathrm{s}}$ to approach the conventional velocity equipartition relation. A comparison shows that the experimentally obtained symbols in Fig. 3 are exactly located on the theoretically predicted dashed line $L(f_{\mathrm{s}})$ in a modified equipartition theorem^24^

4 $$\begin{aligned} \frac{m\langle {\bar{v}}_{f_{\mathrm{s}}}^{2}\rangle }{k_{\mathrm{B}}T}=L(f_{\mathrm{s}}), \end{aligned}$$

where *T* in our experiment is $T_{\mathrm{kin}}$. Here,

5 $$\begin{aligned} L(f_{\mathrm{s}})=2f_{\mathrm{s}}^2\left[ \frac{1}{f_0^2}+\frac{e^{-\frac{f_p+2f_1}{2f_{\mathrm{s}}}}}{f_1 (f_p+2f_1)}-\frac{e^{-\frac{f_p-2f_1}{2f_{\mathrm{s}}}}}{f_1 (f_p-2f_1)}\right] \end{aligned}$$

is used to quantify how seriously the conventional equipartition theorem is violated, where $f_{\mathrm{p}}=\gamma /(2\pi m)$, $f_{\mathrm{k}}=k/(2\pi \gamma )$, $f_{0}=\sqrt{f_{\mathrm{p}}f_{\mathrm{k}}}$, and $f_1=\sqrt{f_{\mathrm{p}}^2/4-f_0^2}$. Notably, no theory–experiment discrepancy is seen in Fig. 3, even when $f_{\mathrm{s}}$ is as large as $10^4$ Hz at 2000 K, which contrasts with the apparent discrepancy under an electric noise^24^. That discrepancy is hypothesized to be a consequence of the concentration-polarization process, in which the electrophoretic response of the Brownian particle is suppressed by the rearrangement of counterions in the electric double layer^24^. Since our Brownian particle is under an optical noise, it does not suffer from such counterion problem and can respond more instantly to that optical stochastic force. This leads to an invisible discrepancy in Fig. 3, which echoes the hypothesis in Ref.^24^.

### Thermodynamic energetics under a varying potential and temperature

**Figure 4:**
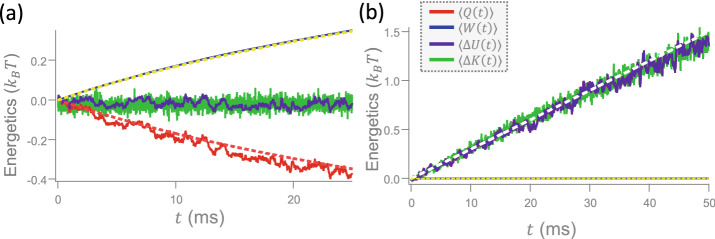
The cumulative sums of various energetic quantities during (a) an iso-*T* compression, where $k_{\mathrm{eff}}$ increases linearly from 10 pN/$\mu$m to 20 pN/$\mu$m within $\tau =25$ ms at $T_{\mathrm{eff}}=300$ K, and (b) an iso-*k* expansion, where $T_{\mathrm{eff}}$ increases linearly from 300 K to 1200 K within $\tau =50$ ms at $k_{\mathrm{eff}}=20$ pN/$\mu$m. In (a), the experimentally attained $\langle Q(t)\rangle$ (red zigzag), $\langle W(t)\rangle$ (blue zigzag), $\langle \Delta U(t)\rangle$ (purple zigzag), and $\langle \Delta K(t)\rangle$ (green zigzag) are compared with the theoretically derived $\langle Q(t)\rangle$ (magenta smooth dashed) and $\langle W(t)\rangle$ (yellow smooth dashed). In this scale, the fluctuation of the blue zigzag line is so small that it looks identical with the yellow smooth dashed line. In (b), the meanings of different lines are the same as those in (a), except for the white dashed line, which replaces the magenta dashed line. Notably, the red line in this plot is hidden under the purple line. All experimentally extracted lines have been averaged over an ensemble of $10^4$ stochastic trajectories. For the calculation of $\langle \Delta K(t)\rangle$, the sampling time for evaluating the velocity is $t_{\mathrm{s}}=100$ $\mu$s.

Before demonstrating the microscopic Stirling engine, we scrutinize the nonequilibrium fluctuations of the Brownian particle in a VP whose effective stiffness, $k_{\mathrm{eff}}$, and temperature, $T_{\mathrm{eff}}$, vary in time, in the regime where $k_{\mathrm{eff}}=k_{\mathrm{v}}$ and $T_{\mathrm{eff}}=T_{\mathrm{kin}}$ in Fig. 2d,h. To determine the energetics of the particle moving along a stochastic trajectory during a given process, we adopt the framework of stochastic thermodynamics^26^. Therein, *Q* (*W*) is the energy transferred by heat (work) from the environment to that particle and the internal energy only contains the potential energy *U* for an overdamped system. While the heat $Q(t)=\int _{x(0)}^{x(t)}({\partial U(k,x)}/{\partial x})dx$, work $W(t)=\int _0^t(dk/dt')(\partial U(k,x)/\partial k)dt'$, and potential energy change $\Delta U(t)=U(t)-U(0)$ can be measured along a trajectory, the kinetic energy change, $\Delta K(t)$, is basically unattainable due to the difficulty of tracing the instantaneous velocity of a fluctuating particle. However, the average kinetic energy change, $\langle \Delta K(t)\rangle$, over an ensemble of systems (particles) can be evaluated from the change of the variance, $\Delta \langle {\bar{v}}_{f_{\mathrm{s}}}^2(t)\rangle$, of the velocity ${\bar{v}}_{f_{\mathrm{s}}}$ via the identity $\langle \Delta K(t)\rangle =\frac{1}{2}m\Delta \langle {\bar{v}}_{f_{\mathrm{s}}}^2(t)\rangle /L(f_{\mathrm{s}})$^24^. In practice, if we define $\Delta K(t)\equiv \frac{1}{2}m\Delta {\bar{v}}_{f_{\mathrm{s}}}^2(t)/L(f_{\mathrm{s}})$ for each trajectory, its ensemble average over all trajectories is exactly the same as the above $\langle \Delta K(t)\rangle$. Although this $\Delta K(t)$ is only an auxiliary quantity lacking of a physical meaning, for convenience we will regard it as an “effective" cumulative sum of kinetic energy change along a trajectory. While the change of the state variable *U*(*t*), or *K*(*t*), only depends on the two end positions of a trajectory, *Q*(*t*) and *W*(*t*) are functions of all intermediate positions along the trajectory. For the experimentally measured particle positions $x_0,\,x_{t_u},\,x_{2t_u},\,...,\,x_{Nt_u}$ within time *t*, the work is calculated by $W(t)\approx (k(t)-k(0))/(2N)\sum _{i=1}^N[(x_{(i-1)t_u}+x_{it_u})/2]^2$ and the heat *Q*(*t*) is evaluated using a Simpson-like quadrature formula^27^.

Figure 4 shows the ensemble-averages of the above cumulative sums of energetics during an iso-*T* compression and an iso-*k* expansion. In both cases, the process is carried out slowly enough for the system to be considered as being in the quasi-static limit. For the iso-*T* compression in Fig. 4a, the experimentally obtained average heat $\langle Q(t)\rangle$ (work $\langle W(t)\rangle$) decreases (increases) logarithmically, as predicted by the analytical formula $\langle Q(t)\rangle =-\langle W(t)\rangle =-k_{\mathrm{B}}T_{\mathrm{eff}}\ln \sqrt{k_{\mathrm{eff}}(t)/k_{\mathrm{eff}}(0)}$ depicted by the magenta (yellow) dashed line.^23^. The average potential energy change, $\langle \Delta U(t)\rangle$, is close to zero, which, together with the above work and heat, agrees with the first law of thermodynamics. The average kinetic energy change, $\langle \Delta K(t)\rangle$, does not vary much in the whole process, which is in consistent with the $k_{\mathrm{v}}$-independent velocity PDFs in Fig. 2b. For the iso-*k* expansion in Fig. 4b, the experimentally attained $\langle Q(t)\rangle$ agrees with the theoretical value $\langle Q(t)\rangle =k_{\mathrm{B}}[T_{\mathrm{eff}}(t)-T_{\mathrm{eff}}(0)]/2$, denoted by the white dashed line. $\langle W(t)\rangle$ stays close to zero, which is plausible because the shape of the VP is never changed during the whole process. $\langle \Delta U(t)\rangle$ overlaps with $\langle Q(t)\rangle$, in consistent with the first law of thermodynamics. Lastly, the linearly increasing $\langle \Delta K(t)\rangle$ coincides with the equipartition theorem confirmed in Fig. 2f.

### A microscopic Stirling engine

**Figure 5:**
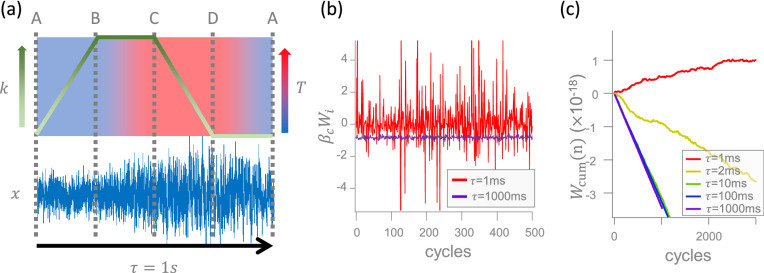
(**a**) (Top panel) A schematic drawing of the single cycle of a microscopic Stirling engine. The white-green arrow denotes the increase of the stiffness, $k_{\mathrm{v}}=k_{\mathrm{eff}}$, of the virtual harmonic potential from $k_{\mathrm{min}}=10$ pN/$\mu$m to $k_{\mathrm{max}}=20$ pN/$\mu$m and the blue-red arrow represents the increase of the kinetic temperature, $T_{\mathrm{kin}}=T_{\mathrm{eff}}$, from $T_{\mathrm{c}}=300$ K to $T_{\mathrm{h}}=1000$ K. In process A$\rightarrow$B (iso-*T* compression), $k_{\mathrm{eff}}$ rises linearly from $k_{\mathrm{min}}$ to $k_{\mathrm{max}}$ at $T_{\mathrm{c}}$. In process B$\rightarrow$C (iso-*k* expansion), $T_{\mathrm{eff}}$ rises linearly from $T_{\mathrm{c}}$ to $T_{\mathrm{h}}$ at $k_{\mathrm{max}}$. In process C$\rightarrow$D (iso-*T* expansion), $k_{\mathrm{eff}}$ falls linearly from $k_{\mathrm{max}}$ to $k_{\mathrm{min}}$ at $T_{\mathrm{h}}$. In process D$\rightarrow$A (iso-*k* compression), $T_{\mathrm{eff}}$ falls linearly from $T_{\mathrm{h}}$ to $T_{\mathrm{c}}$ at $k_{\mathrm{min}}$. (Bottom panel) A trajectory of the Brownian particle during a single cycle. The magnitude of its position fluctuations depends on the values of $k_{\mathrm{eff}}$ and $T_{\mathrm{eff}}$. (**b**) Work fluctuations during 500 cycles of cycle time $\tau =1$ ms (red) and 1s (purple). (**c**) The cumulative sums of work (the integrals of the curves in (**b**)) for various $\tau$.

With all relations justified above, let us move on to the microscopic Stirling engine^10,23^, in which a Brownian particle is manipulated under a time-varying VT and VP, $U_{\mathrm{v}}(x,t)=\frac{1}{2}k_{\mathrm{eff}}(t)x^2$, to convert thermal and artificial fluctuations into mechanical work, in the regime of $k_{\mathrm{eff}}=k_{\mathrm{v}}$ and $T_{\mathrm{eff}}=T_{\mathrm{kin}}$ again as in Fig. 4. In the following, *k* and *T* stand for the above-mentioned stiffness $k_{\mathrm{eff}}$ between its minimum and maximum magnitudes, $[k_{\mathrm{min}},k_{\mathrm{max}}]$, and temperature $T_{\mathrm{eff}}$ between its coldest and hottest values, $[T_{\mathrm{c}},T_{\mathrm{h}}]$, respectively. Figure 5a shows the protocol of our engine (top panel) and a single particle trajectory, *x*(*t*), recorded within a cycle time $\tau =1$ s (bottom panel). The Stirling cycle consists of four processes in the stiffness-temperature space, with the same time span, $\tau /4$, for each process. In the iso-*T* compression, the increase of *k* suppresses the fluctuations of *x*. In the iso-*k* expansion, the increase of *T* raises the fluctuations. In the iso-*T* expansion, the decrease of *k* further enhances the fluctuations. In the iso-*k* compression, the decrease of *T* yields a rapid drop of the fluctuation magnitude to its initial level at the beginning of the cycle.

**Figure 6:**
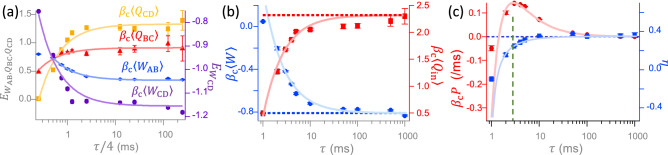
Energetics of the microscopic Stirling engine. (**a**) The experimentally attained $\beta_{\mathrm{c}}\langle W_{\mathrm{AB}}\rangle$ (blue diamonds), $\beta_{\mathrm{c}}\langle W_{\mathrm{CD}}\rangle$ (purple circles), $\beta_{\mathrm{c}}\langle Q_{\mathrm{BC}}\rangle$ (red triangles), and $\beta_{\mathrm{c}}\langle Q_{\mathrm{CD}}\rangle$ (yellow squares) are fitted by four functions of the form $f(\tau )=\pm 1/\tau +\mathrm{const.}$, where the values of $\beta_{\mathrm{c}}\langle W_{\mathrm{AB}}\rangle$, $\beta_{\mathrm{c}}\langle Q_{\mathrm{BC}}\rangle$, and $\beta_{\mathrm{c}}\langle Q_{\mathrm{CD}}\rangle$ ($\beta_{\mathrm{c}}\langle W_{\mathrm{CD}}\rangle$) are presented on the left (right) axis labeled by $E_{W_{\mathrm{AB}}, Q_{\mathrm{BC}}, Q_{\mathrm{CD}}}$ ($E_{W_{\mathrm{CD}}}$). Therein, the blue line is not connected to the leftmost blue point, because work does not obey the above $1/\tau$ relation in this small $\tau$ regime, as observed in several of our experiments. (**b**) The dimensionless average total work, $\beta_{\mathrm{c}}\langle W\rangle =\beta_{\mathrm{c}}(\langle W_{\mathrm{AB}}\rangle +\langle W_{\mathrm{CD}}\rangle )$, (red circles) and injected heat, $\beta_{\mathrm{c}}\langle Q\rangle =\beta_{\mathrm{c}}(\langle Q_{\mathrm{BC}}\rangle +\beta_{\mathrm{c}}\langle Q_{\mathrm{CD}}\rangle )$, (blue squares) over a cycle are fitted by two functions of the same form as $f(\tau )$. (**c**) The rescaled power output, $\beta_{\mathrm{c}}P=\beta_{\mathrm{c}}\langle W\rangle /\tau$, (red circles and line) and the engine efficiency, $\eta =-\langle W\rangle /\langle Q\rangle$, (blue squares and line), where symbols are calculated from the data in (**b**) and the lines are given by Eqs. (6) and (7), respectively. The power has a peak at $\tau \approx 2.8$ ms (green dashed line and the efficiency saturates at its maximum, $\eta _{\infty }\approx 0.35$, (horizontal blue dashed line) in the quasi-static limit $\tau \rightarrow \infty$. The errors in all symbols from (**a**) to (**c**) are the 95$\%$ confidence interval.

Figure 5b depicts the fluctuations of the dimensionless work $\beta_{\mathrm{c}}W_i$ in the *i*-th cycle along a trajectory of 500 cycles, where $\beta_{\mathrm{c}}\equiv 1/(k_{\mathrm{B}}T_{\mathrm{c}})$ denotes an inverse temperature. For a smaller cycle time, such as $\tau =1$ ms, the work fluctuates intensively between negative and positive values and has a positive average. For a larger cycle time, such as $\tau =1$ s, the fluctuations become weaker and negative in any cycle. The corresponding cumulative sum of work $W_{\mathrm{cum}}(n)=\sum _{i=1}^{n}W_i$ within *n* cycles are shown in Fig. 5c. The decline of $W_{\mathrm{cum}}(n)$ characterizes the typical capability of a heat engine to do work on the surrounding. However, for $\tau =1$ ms (red curve), the increase of $W_{\mathrm{cum}}(n)$ indicates that the surrounding will do work on the system. Thus, the cycle time can serve as a parameter to switch the direction of the energy flow of this microscopic system. The critical cycle time for the sign change of the work production in Fig. 5c is $\tau \approx$ 1.4 ms, which can be evaluated from the blue curve of work in Fig. 6b.

In Fig. 6a, our experiment shows that all the observed dimensionless average work and heat in different processes obey a similar inverse relation to $\tau$: $\beta_{\mathrm{c}}\langle W_{\mathrm{AB}}\rangle =\beta_{\mathrm{c}}\langle W_{\mathrm{AB}}\rangle _{\infty }+4A_{\mathrm{AB}}/\tau$, $\beta_{\mathrm{c}}\langle W_{\mathrm{CD}}\rangle =\beta_{\mathrm{c}}\langle W_{\mathrm{CD}}\rangle _{\infty }+4A_{\mathrm{CD}}/\tau$, $\beta_{\mathrm{c}}\langle Q_{\mathrm{BC}}\rangle =\beta_{\mathrm{c}}\langle Q_{\mathrm{BC}}\rangle _{\infty }-4B_{\mathrm{BC}}/\tau$, and $\beta_{\mathrm{c}}\langle Q_{\mathrm{CD}}\rangle =\beta_{\mathrm{c}}\langle Q_{\mathrm{CD}}\rangle _{\infty }-4B_{\mathrm{CD}}/\tau$, with $A_{\mathrm{AB}}=0.19\pm 0.004$ ms, $A_{\mathrm{CD}}=0.10\pm 0.004$ ms, $B_{\mathrm{BC}}=0.34\pm 0.023$ ms, and $B_{\mathrm{CD}}=0.10 \pm 0.012$ ms. Here, $\langle \cdot \rangle _{\infty }$ represents an ensemble average in the quasi-static limit ($\tau \rightarrow \infty$), $4/\tau$ arises from the time $\tau /4$ for each of the four processes in a cycle, and $A_{\mathrm{CD}}\approx B_{\mathrm{CD}}$ can be explained by the first law of thermodynamics. The experimentally observed $1/\tau$ dependence of $\langle W_{\mathrm{AB}}\rangle$, $\langle W_{\mathrm{CD}}\rangle$, and $\langle Q_{\mathrm{CD}}\rangle$ for the iso-*T* process agree with other theoretical analysis^22,28^, while that of $\langle Q_{\mathrm{BC}}\rangle$ for an iso-*k* process has, to the best of our knowledge, never been reported before. This experimentally observed $\langle Q_{\mathrm{BC}}\rangle$ resembles the low-dissipation form of $\langle W_{\mathrm{AB}}\rangle$ and $\langle W_{\mathrm{CD}}\rangle$ known in the iso-*T* processes^22,29,30^ and requires a further theoretical analysis.

Summing up individual values of work (heat) in Fig. 6a, one gets the dimensionless total extracted work, $\beta_{\mathrm{c}}\langle W\rangle =\beta_{\mathrm{c}}(\langle W_{\mathrm{AB}}\rangle +\langle W_{\mathrm{CD}}\rangle )$ (injected heat, $\beta_{\mathrm{c}}\langle Q\rangle =\beta_{\mathrm{c}}(\langle Q_{\mathrm{BC}}\rangle +\langle Q_{\mathrm{CD}}\rangle )$), during a cycle in Fig. 6b. Taking the analytical expressions $\beta_{\mathrm{c}}\langle W_{\mathrm{AB}}\rangle _{\infty }=\ln \sqrt{a}$, $\beta_{\mathrm{c}}\langle W_{\mathrm{CD}}\rangle _{\infty }=-(T_h/T_c)\ln \sqrt{a}$, $\beta_{\mathrm{c}}\langle Q_{\mathrm{BC}}\rangle _{\infty }=(T_h/T_c-1)/2$, and $\beta_{\mathrm{c}}\langle Q_{\mathrm{CD}}\rangle _{\infty }=(T_h/T_c)\ln \sqrt{a}$ from Ref.^23^, it yields $\beta_{\mathrm{c}}\langle W\rangle =\left[ 1-T_{\mathrm{h}}/T_{\mathrm{c}}\right] \ln \sqrt{a}+A/\tau$ and $\beta_{\mathrm{c}}\langle Q\rangle =-(1/2)\left[ 1-T_{\mathrm{h}}/T_{\mathrm{c}}\right] +(T_{\mathrm{h}}/T_{\mathrm{c}})\ln \sqrt{a}-B/\tau$, where $a=k_{\mathrm{max}}/k_{\mathrm{min}}$. Here, $A\equiv 4(A_{\mathrm{AB}}+A_{\mathrm{CD}})=1.16\pm 0.09$ ms and $B\equiv 4(B_{\mathrm{BC}}+B_{\mathrm{CD}})=1.89\pm 0.20$ ms for the values of $k_{\mathrm{max}}$, $k_{\mathrm{min}}$, $T_{\mathrm{h}}$, and $T_{\mathrm{c}}$ assigned in Fig. 5a. In Fig. 6b, the dimensionless average work, $\beta_{\mathrm{c}}\langle W\rangle$, is positive at $\tau =1$ ms, decreases monotonically with the increasing $\tau$, and converges to a negative value at $\beta_{\mathrm{c}}\langle W\rangle _\infty \equiv \beta_{\mathrm{c}}(\langle W_{\mathrm{AB}}\rangle _\infty +\langle W_{\mathrm{CD}}\rangle _\infty )=-0.81$, in the quasi-static limit. By contrast, the dimensionless average heat, $\beta_{\mathrm{c}}\langle Q\rangle$, is always positive regardless of the cycle time, rises monotonically, and approaches asymptotically the theoretical value at $\beta_{\mathrm{c}}\langle Q\rangle _\infty \equiv \beta_{\mathrm{c}}(\langle Q_{\mathrm{BC}}\rangle _\infty +\langle Q_{\mathrm{CD}}\rangle _\infty )=2.32$.

To evaluate the performance of the engine, we calculate two engine characteristics, the power output and the efficiency. The former is the work done by the engine per cycle time, which after being rescaled by $\beta_{\mathrm{c}}^{-1}$ is equal to

6 $$\begin{aligned} \beta_{\mathrm{c}}P=-\beta_{\mathrm{c}}\langle W\rangle /\tau =-\beta_{\mathrm{c}}\langle W\rangle _{\infty }/\tau -A/\tau ^2. \end{aligned}$$

The efficiency of the microscopic Stirling engine is the ratio of the extracted work to the injected heat,

7 $$\begin{aligned} \eta (\tau )&=-\frac{\langle W\rangle }{\langle Q\rangle } =\frac{\eta_{\mathrm{c}} - 2AT_{\mathrm{c}}/(T_{\mathrm{h}}\tau \ln a) }{\eta_{\mathrm{c}}/\ln a+1-2BT_{\mathrm{c}}/(T_{\mathrm{h}}\tau \ln a)}, \end{aligned}$$

with $\eta_{\mathrm{c}}\equiv 1-T_{\mathrm{c}}/T_{\mathrm{h}}$ the Carnot efficiency. In the quasi-static limit, inserting $\tau \rightarrow \infty$ into Eq. (7) will give the limiting efficiency^23^

8 $$\begin{aligned} \eta _\infty =\frac{\eta_{\mathrm{c}}}{1+\eta_{\mathrm{c}}/\ln a}. \end{aligned}$$

This efficiency is always lower than the Carnot efficiency due to the additional heat input during the iso-*k* expansion. If we replace the stiffness ratio *a* by the volume compression ratio, Eq. (8) will recover the efficiency of the macroscopic Stirling engine (without a regenerator to store the output heat).

In Fig. 6c, the measured $\beta_{\mathrm{c}}P$ (red circles) lie rather close to the theoretical curve (red line) given by Eq. (6) with the *A* there taken from Fig. 6b. The measured efficiency (blue squares) are almost located directly on the theoretical curve (blue line) determined from Eq. (7). The blue line increases monotonically from a negative number, where $\tau$ is smaller than the intrinsic relaxation time $t_{\mathrm{R}}=\gamma /k_{\mathrm{min}}\approx 1$ ms, and asymptotically converges to its maximum value $\eta _\infty$ in Eq. (8) in the quasi-static limit when $\tau >50$ ms. The convergence process within milliseconds is much faster than that within seconds in a previous study^10^. Since $T_{\mathrm{v}}$ in our system can be more instantly tuned than the water temperature heated by laser in that study, we can more easily access systems far beyond the quasi-static regime.

As commonly known and seen in Fig. 6c, there exists a trade-off relation between the efficiency and power, where the latter will become negligibly small when the former arrives at its maximum at large $\tau$. The maximum power, $P^*$, is determined by the zero of the derivative of Eq. (6) with respect to $\tau$. The cycle time at that $P^*$ is $\tau ^*=4A/[(T_{\mathrm{h}}/T_{\mathrm{c}}-1)\ln a]$, as depicted by the green dashed line in Fig. 6c. Inserting $\tau ^*$ into the $\tau$ of the above $\langle W\rangle$ and $\langle Q\rangle$, one obtains $\langle Q^*\rangle$ and $\langle W^*\rangle$, respectively, and subsequently the efficiency at maximum power (EMP) of the microscopic Stirling engine,

9 $$\begin{aligned} \eta ^*=-\frac{\langle W^*\rangle }{\langle Q^*\rangle }=\frac{\eta_{\mathrm{c}}}{2-{\tilde{\alpha }}\eta_{\mathrm{c}}+2\eta_{\mathrm{c}}/\ln a}, \end{aligned}$$

where ${\tilde{\alpha }}\equiv B/A$. For the values of *A* and *B* mentioned before Eq. (6), the above formulas imply ${\tilde{\alpha }}\approx 1.63$, $\tau ^*\approx 2.8$ ms, $\beta_{\mathrm{c}}P^*\approx 0.14$ ms$^{-1}$, and $\eta ^*\approx 0.24$. Taking a series expansion of the EMP in Eq. (9), we obtain $\eta ^*=\eta_{\mathrm{c}}/({2-{\tilde{\alpha }}\eta_{\mathrm{c}}+2\eta_{\mathrm{c}}/\ln a})=(1/2)\eta_{\mathrm{c}}+({\tilde{\alpha }}/4-1/(2\ln {a}))\eta_{\mathrm{c}}^2+O(\eta_{\mathrm{c}}^3)$. It has a linear term 1/2, which follows the model-independent universal EMP property in the linear response regime^28,30^.

It is interesting to compare the EMP of our Stirling engine with those of other Carnot engines. Notice that the EMP pointed by the green dashed line in Fig. 6c is calculated under the condition of equal durations, $\tau _{\mathrm{AB}}=\tau _{\mathrm{BC}}=\tau _{\mathrm{CD}}=\tau _{\mathrm{DA}}=\tau /4$. It is stricter than the commonly derived EMP without a specified duration relation, as in Ref.^28^. For general engines, the EMP is bounded by $\eta _{\mathrm{CA}}=1-\sqrt{T_{\mathrm{c}}/T_{\mathrm{h}}}=1-\sqrt{1-\eta_{\mathrm{c}}}$, as proposed by Curzon and Ahlborn in the framework of finite-time thermodynamics^31^. For the specific stochastic Carnot engine in Ref.^22^, its EMP, ${\bar{\eta }}^*=\eta_{\mathrm{c}}/[2-{\bar{\alpha }}\eta_{\mathrm{c}}]$, looks similar to our ${\eta }^*$ in Eq. (9), with ${\bar{\alpha }}$ the irreversible action. Since ${\bar{\alpha }}$ can be explicitly related to the stiffness and temperature in that analytically solvable model, one can discuss whether ${\bar{\eta }}^*$ is bounded by $\eta _{\mathrm{CA}}$ for certain parameter range or not. For general engines, unfortunately, that question does not have an analytical answer and can only resort to experiments or simulations. The empirical value of ${\tilde{\alpha }}$ extracted from Fig. 6a provides an explicit example showing a relation between $\eta ^*\approx 0.24$, $\eta _{\mathrm{CA}}= 0.45$, and $\eta_{\mathrm{c}}=0.70$.

## Discussion

With the recent active development in the thermodynamics for small systems, seeking convenient and accurate experimental techniques to realize and test concepts in this field becomes increasingly in demand. This work demonstrates how to use a single laser beam to simultaneously control heat and work to realize a microscopic heat engine. The results not only justify the feasibility of this idea but also feature its high accuracy. Since the VP and VT in this technique can be flexibly changed, one can readily modify them to mimic confining potentials beyond harmonic shapes and various stochastic forces, including colored and non-Gaussian noises. With these merits, the current study opens an attractive possibility for exploring diverse energetics related questions in the small world. Some recent examples include systems affected by active heat baths^11,32,33^ and the high-efficient heat engines sped up by shortcuts-to-adiabaticity protocols^34^.

## Supplementary Information

Supplementary Information.
