# Supplementary material for: Shift a laser beam back and forth to exchange heat and work in thermodynamics
Source: Sci Rep. 2021 Feb 23;11:4394. doi: 10.1038/s41598-021-83824-7 (PMC7902854; doi:10.1038/s41598-021-83824-7)
Supplement: Supplementary file 1 — Supplementary Information. [file 41598_2021_83824_MOESM1_ESM.pdf]

Supplemental Material for "Shift a laser beam back and forth to exchange heat and work in thermodynamics"

John A. C. Albay[1], Zhi-Yi Zhou[1], Cheng-Hung Chang[2],* and Yonggun Jun[1†]

[1]*Department of Physics, National Central University, Taoyuan, 320, Taiwan*

[2]*Institute of Physics, National Chiao Tung University, Hsinchu, 300, Taiwan*

(Dated: January 25, 2021)

* Electronic Address: chchang@mail.nctu.edu.tw

† Electronic Address: yonggun@phy.ncu.edu.tw

Here we collect some basic calculations for the particle dynamics in the optical feedback trap in the main text. More general treatments are referred to the literatures of stochastic processes. More discussions about numerical error control can be found in Ref. [1], e.g., 4.1.2.5 Numerical Precision Required for the Energetics.

A. Langevin equation for two noises

Let us consider the Langevin equation in Sec. II of the main text

$$\gamma \dot{x} + kx = \xi_v^f + \xi^f$$

and its alternative expression

$$\dot{x} + \frac{k}{\gamma}x = \frac{\xi_v^f}{\gamma} + \frac{\xi^f}{\gamma}, \tag{S1}$$

where the thermal noise ξ^f and the external noise ξ_v^f are Gaussian and white, with

$$\begin{aligned} \langle \xi^f(t)\xi^f(t')\rangle &= A\delta(t-t') \\ \langle \xi_v^f(t)\xi_v^f(t')\rangle &= A_v\delta(t-t') \\ \langle \xi_v^f(t)\xi^f(t')\rangle &= 0 \qquad \text{for all } t \text{ and } t'. \end{aligned} \tag{S2}$$

Following a standard derivation, the particle dynamics starting at $x(0) = 0$ is

$$x(t) = \frac{1}{\gamma}e^{-\frac{k}{\gamma}t}\int_0^t e^{\frac{k}{\gamma}s}\left(\xi_v^f(s) + \xi^f(s)\right)ds,$$

Its correlation is (without loss of generality, we assume $t' \le t$)

$$\begin{aligned} \langle x(t)x(t')\rangle &= \frac{1}{\gamma^2}e^{-\frac{k}{\gamma}(t+t')}\int_0^{t'}\int_0^t e^{\frac{k}{\gamma}(s+s')}(\langle \xi_v^f(s)\xi_v^f(s')\rangle + \langle \xi^f(s)\xi^f(s')\rangle)dsds' \\ &= \frac{1}{\gamma^2}e^{-\frac{k}{\gamma}(t+t')}\int_0^{t'}\int_0^t e^{\frac{k}{\gamma}(s+s')}(A+A_v)\underbrace{\delta(s-s')ds}_{\text{dim of time}}ds' \\ &= \frac{A+A_v}{\gamma^2}e^{-\frac{k}{\gamma}(t+t')}\underbrace{\int_0^{t'} e^{2s'\frac{k}{\gamma}}ds'}_{\text{time}}\cdot\underbrace{1}_{\text{time}} \\ &= \frac{A+A_v}{\gamma^2}e^{-\frac{k}{\gamma}(t+t')}\left[\frac{\gamma}{2k}e^{2s'\frac{k}{\gamma}}\right]_0^{t'}\cdot\underbrace{1}_{\text{time}} \\ &= \frac{A+A_v}{2k\gamma}e^{-\frac{k}{\gamma}(t+t')}\left(e^{2\frac{k}{\gamma}t'}-1\right)\cdot\underbrace{1}_{\text{time}} \\ &= \frac{\widetilde{A}+\widetilde{A}_v}{2k\gamma}e^{-\frac{k}{\gamma}(t+t')}\left(e^{2\frac{k}{\gamma}t'}-1\right), \end{aligned}$$

where $\widetilde{A}+\widetilde{A}_v \equiv (A+A_v)\cdot\underbrace{1}_{\text{time}}$ has a unit of "force^2·time". Throughout this calculation, we keep ·1 and /1 to remind the correct dimension. For $t'=t$,

$$\langle x^2(t)\rangle = \frac{\widetilde{A}+\widetilde{A}_v}{2k\gamma}\left(1-e^{-2\frac{k}{\gamma}t}\right). \tag{S3}$$

Two special cases of Eq. (S3):

1. For a free particle ($k\to 0$) at $t<\infty$,

$$\begin{aligned}\langle x^2(t)\rangle =& \frac{\widetilde{A}+\widetilde{A}_v}{2k\gamma}\left(1-e^{-2\frac{k}{\gamma}t}\right)\\ \xrightarrow{k\to 0(L'\text{Hospital rule})}& \frac{\widetilde{A}+\widetilde{A}_v}{2\gamma}\left(\frac{2t}{\gamma}e^{-2\frac{k}{\gamma}t}\right)_{k=0} = \frac{\widetilde{A}+\widetilde{A}_v}{\gamma^2}t \equiv 2Dt.\end{aligned}$$

   Here, the noise strength, $A+A_v$, has been expressed as a "diffusion constant" D through the relation

$$(A+A_v)\cdot\underbrace{1}_{\text{time}} = \widetilde{A}+\widetilde{A}_v \equiv 2\gamma^2 D.$$

2. For a confined particle ($k>0$) at $t\to\infty$, Eq. (S3) becomes

$$\langle x^2(t)\rangle = \frac{\widetilde{A}+\widetilde{A}_v}{2k\gamma}\left(1-e^{-2\frac{k}{\gamma}t}\right) \xrightarrow{t\to\infty} \frac{\widetilde{A}+\widetilde{A}_v}{2k\gamma}. \tag{S4}$$

   For $\xi_v^f(t)=\widetilde{A}_v=0$, using the equipartition theorem, $\langle x^2(t)\rangle = \frac{k_BT}{k}$, Eq. (S4) implies

$$\widetilde{A} = 2\gamma k_B T = A\cdot 1. \tag{S5}$$

   For $\xi^f(t)=\widetilde{A}=0$, assuming an analogous relation $\langle x^2(t)\rangle = \frac{k_BT_v}{k}$, one can define an artificial temperature T_V for ξ_v^f by

$$\widetilde{A}_v = 2\gamma k_B T_V = A_v\cdot 1. \tag{S6}$$

   As a result, the correlation functions Eq. (S2) can be reexpressed as

$$\langle\xi^f(t)\xi^f(t')\rangle = A\delta(t-t') = \frac{2\gamma k_BT}{1}\cdot\delta(t-t') \tag{S7}$$
$$\langle\xi_v^f(t)\xi_v^f(t')\rangle = A_v\delta(t-t') = \frac{2\gamma k_BT_V}{1}\cdot\delta(t-t')$$

   with

$$\xi^f(t) = \sqrt{\frac{2\gamma k_BT}{1}}g(1) \tag{S8}$$
$$\xi_v^f(t) = \sqrt{\frac{2\gamma k_BT_V}{1}}g(1), \tag{S9}$$

   where $g(1)$ is a Gaussian white noise of zero mean and unite variance.

B. A difference equation of the Langevin equation

Equation (S1) has a difference equation (See Sekimoto for more general cases)

$$\frac{x_{t+\Delta} - x_t}{\Delta} + \frac{k}{\gamma} x_t = \left(\widetilde{\xi}_v^f + \widetilde{\xi}^f \right) \frac{1}{\gamma}, \tag{S10}$$

with two random variables $\widetilde{\xi}_v^f$ and $\widetilde{\xi}^f$ to be determined and a short time-span Δ. The particle dynamics following this equation is

$$x_{t+\Delta} = \left(1 - \Delta \frac{k}{\gamma} \right) x_t + \left(\widetilde{\xi}_v^f + \widetilde{\xi}^f \right) \frac{\Delta}{\gamma} \equiv \Omega x_t + \left(\widetilde{\xi}_v^f + \widetilde{\xi}^f \right) \frac{\Delta}{\gamma}, \tag{S11}$$

where $\Omega \equiv 1 - \frac{\Delta}{\tau_R}$, with $\tau_R \equiv \frac{\gamma}{k}$. The iteration relation in Eq. (S11) implies

$$\begin{cases} x_{\Delta} = \Omega x_0 + \left(\widetilde{\xi}_{v,1}^f + \widetilde{\xi}_1^f \right) \frac{\Delta}{\gamma} \\ x_{2\Delta} = \Omega x_{\Delta} + \left(\widetilde{\xi}_{v,2}^f + \widetilde{\xi}_2^f \right) \frac{\Delta}{\gamma} \\ x_{3\Delta} = \Omega x_{2\Delta} + \left(\widetilde{\xi}_{v,3}^f + \widetilde{\xi}_3^f \right) \frac{\Delta}{\gamma} \\ \vdots \\ x_{(N-1)\Delta} = \Omega x_{(N-2)\Delta} + \left(\widetilde{\xi}_{v,(N-1)}^f + \widetilde{\xi}_{(N-1)}^f \right) \frac{\Delta}{\gamma} \\ x_{N\Delta} = \Omega x_{(N-1)\Delta} + \left(\widetilde{\xi}_{v,N}^f + \widetilde{\xi}_N^f \right) \frac{\Delta}{\gamma}. \end{cases}$$

Multiplying these equations by proper Ω^n , one obtains

$$\begin{cases} \Omega^{N-1} x_{\Delta} = \Omega^N x_0 + \left(\widetilde{\xi}_{v,1}^f + \widetilde{\xi}_1^f \right) \frac{\Delta}{\gamma} \Omega^{N-1} \\ \Omega^{N-2} x_{2\Delta} = \Omega^{N-1} x_{\Delta} + \left(\widetilde{\xi}_{v,2}^f + \widetilde{\xi}_2^f \right) \frac{\Delta}{\gamma} \Omega^{N-2} \\ \Omega^{N-3} x_{3\Delta} = \Omega^{N-2} x_{2\Delta} + \left(\widetilde{\xi}_{v,3}^f + \widetilde{\xi}_3^f \right) \frac{\Delta}{\gamma} \Omega^{N-3} \\ \vdots \\ \Omega x_{(N-1)\Delta} = \Omega^2 x_{(N-2)\Delta} + \left(\widetilde{\xi}_{v,(N-1)}^f + \widetilde{\xi}_{(N-1)}^f \right) \frac{\Delta}{\gamma} \Omega \\ x_{N\Delta} = \Omega x_{(N-1)\Delta} + \left(\widetilde{\xi}_{v,N}^f + \widetilde{\xi}_N^f \right) \frac{\Delta}{\gamma}. \end{cases}$$

After summing up all these equations, we get the particle position after N steps of duration Δ at time $t = N\Delta$:

$$\begin{aligned} x_{N\Delta} = \Omega^N x_0 + \Big[& \left(\widetilde{\xi}_{v,1}^f \Omega^{N-1} + \widetilde{\xi}_{v,2}^f \Omega^{N-2} + \ldots + \widetilde{\xi}_{v,(N-1)}^f \Omega + \widetilde{\xi}_{v,N}^f \right) \\ & + \left(\widetilde{\xi}_1^f \Omega^{N-1} + \widetilde{\xi}_2^f \Omega^{N-2} + \ldots + \widetilde{\xi}_{(N-1)}^f \Omega + \widetilde{\xi}_N^f \right) \Big] \frac{\Delta}{\gamma} \equiv \Omega^N x_0 + \Xi \frac{\Delta}{\gamma}, \end{aligned} \tag{S12}$$

where Ξ denotes the sum of all terms in the parenthesis $[\ldots]$. For $x_0 = 0$,

$$
\begin{aligned}
\langle x_{N\Delta}^2 \rangle =& \langle \Xi^2 \rangle \frac{\Delta^2}{\gamma^2} \\
=& \Big[\langle (\widetilde{\xi}_{v,1}^f)^2 \rangle \Omega^{2(N-1)} + \langle (\widetilde{\xi}_{v,2}^f)^2 \rangle \Omega^{2(N-2)} + \ldots + \langle (\widetilde{\xi}_{v,(N-1)}^f)^2 \rangle \Omega^2 + \langle (\widetilde{\xi}_{v,N}^f)^2 \rangle \\
&+ \langle (\widetilde{\xi}_1^f)^2 \rangle \Omega^{2(N-1)} + \langle (\widetilde{\xi}_2^f)^2 \rangle \Omega^{2(N-2)} + \ldots + \langle (\widetilde{\xi}_{(N-1)}^f)^2 \rangle \Omega^2 + \langle (\widetilde{\xi}_N^f)^2 \rangle \Big] \frac{\Delta^2}{\gamma^2} \\
=& \Big[\langle (\widetilde{\xi}_v^f)^2 \rangle \left(\Omega^{2(N-1)} + \Omega^{2(N-2)} + \ldots + \Omega^2 + 1 \right) \\
&+ \langle (\widetilde{\xi}^f)^2 \rangle \left(\Omega^{2(N-1)} + \Omega^{2(N-2)} + \ldots + \Omega^2 + 1 \right) \Big] \frac{\Delta^2}{\gamma^2} \\
=& \left[\langle (\widetilde{\xi}_v^f)^2 \rangle + \langle (\widetilde{\xi}^f)^2 \rangle \right] Z \frac{\Delta^2}{\gamma^2},
\end{aligned} \tag{S13}
$$

where

$$
Z \equiv \Omega^{2(N-1)} + \Omega^{2(N-2)} + \ldots + \Omega^2 + 1. \tag{S14}
$$

Two special cases:

1. For a free particle ($k = 0$) and $t < \infty$,

$$
\Omega = 1 - \Delta \frac{k}{\gamma} = 1 \quad \text{and } Z = N,
$$

which gives

$$
\langle x_{N\Delta}^2 \rangle = \left[\langle (\widetilde{\xi}_v^f)^2 \rangle + \langle (\widetilde{\xi}^f)^2 \rangle \right] N \frac{\Delta^2}{\gamma^2} = \left[\langle (\widetilde{\xi}_v^f)^2 \rangle + \langle (\widetilde{\xi}^f)^2 \rangle \right] \frac{\Delta}{\gamma^2} t.
$$

2. For a confined particle ($k > 0$) and $t \to \infty$, Eq. (S14) implies

$$
\Omega^2 Z = \Omega^{2N} + \Omega^{2(N-1)} + \ldots + \Omega^4 + \Omega^2,
$$

$$
\left(\Omega^2 - 1 \right) Z = \Omega^{2N} - 1, \quad \text{and} \quad Z = \frac{1 - \Omega^{2N}}{1 - \Omega^2},
$$

where normally $\Omega = 1 - \Delta \frac{k}{\gamma} \approx 0.9 \ldots < 1$, because $\Delta \frac{k}{\gamma}$ needs to be sufficiently small. Taking $t = N\Delta \to \infty$, it yields

$$
Z = \frac{1 - \Omega^{2N}}{1 - \Omega^2} \xrightarrow{t = N\Delta \to \infty} \frac{1}{1 - \Omega^2}.
$$

Thus, Eq. (S13) becomes

$$
\langle x_{N\Delta}^2 \rangle \xrightarrow{t = N\Delta \to \infty} \left[\langle (\widetilde{\xi}_v^f)^2 \rangle + \langle (\widetilde{\xi}^f)^2 \rangle \right] \left(\frac{1}{1 - \Omega^2} \right) \frac{\Delta^2}{\gamma^2}. \tag{S15}
$$

C. Consistency between differential and difference equations

Since $\langle x^2_{N\Delta}\rangle \xrightarrow{t=N\Delta\to\infty} \left[\langle(\widetilde{\xi}^f_v)^2\rangle + \langle(\widetilde{\xi}^f)^2\rangle\right]\left(\frac{1}{1-\Omega^2}\right)\frac{\Delta^2}{\gamma^2}$ from Eq. (S15) must describe the same particle dynamics as $\langle x^2(t)\rangle \xrightarrow{t\to\infty} \frac{\widetilde{A}+\widetilde{A}_v}{2k\gamma}$ from Eq. (S4), it yields

$$\langle(\widetilde{\xi}^f_v)^2\rangle + \langle(\widetilde{\xi}^f)^2\rangle = \frac{\left(\widetilde{A}+\widetilde{A}_v\right)(1-\Omega^2)\gamma}{2k\Delta^2}.$$

In analogy to the arguments for Eq. (S5) and (S6), setting $\widetilde{\xi}^f_v = \xi^f_v = \widetilde{A}_v = 0$ and $\xi^f = \widetilde{\xi}^f = \widetilde{A} = 0$ individually leads to

$$\langle(\widetilde{\xi}^f)^2\rangle = \frac{\widetilde{A}(1-\Omega^2)\gamma}{2k\Delta^2} \quad \text{and} \quad \langle(\widetilde{\xi}^f_v)^2\rangle = \frac{\widetilde{A}_v(1-\Omega^2)\gamma}{2k\Delta^2}$$

and subsequently

$$\begin{aligned}\widetilde{\xi}^f &= \sqrt{\frac{\widetilde{A}(1-\Omega^2)\gamma}{2k\Delta^2}}g(1)\overbrace{=}^{(S5)} = \frac{\gamma}{\Delta}\sqrt{\frac{k_BT(1-\Omega^2)}{k}}g(1),\\ \widetilde{\xi}^f_v &= \sqrt{\frac{\widetilde{A}_v(1-\Omega^2)\gamma}{2k\Delta^2}}g(1)\overbrace{=}^{(S6)} = \frac{\gamma}{\Delta}\sqrt{\frac{k_BT_v(1-\Omega^2)}{k}}g(1)\end{aligned} \tag{S16}$$

respectively, as in the main text.

For small $\Delta\frac{k}{\gamma}$, $1-\Omega^2 = (1+\Omega)(1-\Omega) = \left(1+1-\Delta\frac{k}{\gamma}\right)\left(1-\left(1-\Delta\frac{k}{\gamma}\right)\right) = \left(2-\Delta\frac{k}{\gamma}\right)\left(\Delta\frac{k}{\gamma}\right) \approx 2\Delta\frac{k}{\gamma}$. Thus, Eq. (S16) will reduce to

$$\begin{aligned}\widetilde{\xi}^f &= \sqrt{\frac{\widetilde{A}}{\Delta}}g(1)\overbrace{=}^{(S5)}\sqrt{\frac{2\gamma k_BT}{\Delta}}g(1),\\ \widetilde{\xi}^f_v &= \sqrt{\frac{\widetilde{A}_v}{\Delta}}g(1)\overbrace{=}^{(S6)}\sqrt{\frac{2\gamma k_BT_v}{\Delta}}g(1),\end{aligned} \tag{S17}$$

which are used in our study, not because we have a small k, but a small Δ.

D. The dynamics for the center of the optical tweezers

Given $\Delta = t_u$ the acquisition time in the main text, the differential equation Eq. (S1),

$$\dot{x} + \frac{k}{\gamma}x - \frac{\xi^f_v}{\gamma} = \frac{\xi^f}{\gamma},$$

with $\xi^f_v \equiv \sqrt{\frac{2\gamma k_BT_v}{1}}g(1)$ and $\xi^f \equiv \sqrt{\frac{2\gamma k_BT}{1}}g(1)$ in Eq. (S9), has a difference equation Eq. (S10),

$$\frac{x_{t+t_u}-x_t}{t_u} + \frac{k}{\gamma}x_t - \frac{\widetilde{\xi}^f_v}{\gamma} = \frac{\widetilde{\xi}^f}{\gamma}, \tag{S18}$$

with $\widetilde{\xi}_v^f \equiv \sqrt{\frac{2\gamma k_B T_V}{t_u}} g(1)$ and $\widetilde{\xi}_t^f \equiv \sqrt{\frac{2\gamma k_B T}{t_u}} g(1)$ in Eq. (S17). Equation (S18) can be rewritten as

$$\frac{x_{t+t_u} - x_t}{t_u} - \frac{1}{\gamma} \underbrace{\left(-kx_t + \widetilde{\xi}_v^f\right)}_{\equiv F_t} = \frac{\widetilde{\xi}^f}{\gamma},$$

where F_t is to be generated by the force

$$f_{ot} = -k_{ot}\left(x_t - x_{L,t}\right) \tag{S19}$$

of an optical tweezers of harmonic shape of stiffness k_{ot} centered at $x_{L,t}$. From $F_t = f_{ot}$ it follows the relation

$$-kx_t + \widetilde{\xi}_v^f = -k_{ot}\left(x_t - x_{L,t}\right) \tag{S20}$$

After a rearrangement, one obtains

$$x_{L,t} = x_t - \frac{k}{k_{ot}} x_t + \frac{\widetilde{\xi}_v^f}{k_{ot}} = -\alpha x_t + \frac{\widetilde{\xi}_v^f}{k_{ot}}, \tag{S21}$$

with $\alpha \equiv -\left(1 - \frac{k}{k_{ot}}\right)$. Taking the delay time t_d into account, Eq. (S21) is modified as

$$x_{L,t} = -\alpha x_{t-t_d} + \frac{\widetilde{\xi}_v^f}{k_{ot}}, \tag{S22}$$

which is Eq. (1) in the main text. Inserting Eq. (S22) into Eq. (S20) gives

$$kx_t = k_{ot}\left(x_t + \alpha x_{t-t_d}\right). \tag{S23}$$

Inserting Eq. (S23) into Eq. (S18) gives

$$\frac{x_{t+t_u} - x_t}{t_u} + \frac{k_{ot}}{\gamma}\left(x_t + \alpha x_{t-t_d}\right) - \frac{\widetilde{\xi}_v^f}{\gamma} = \frac{\widetilde{\xi}^f}{\gamma}, \tag{S24}$$

which is Eq. (2) in the main text.

[1] K. Sekimoto, *Stochastic Energetics*, Lecture Notes in Physics, Vol. 799 (Springer Berlin Heidelberg, Berlin, Heidelberg, 2010).
